# The Serum Lead level in Patients With Retained Lead Pellets

**DOI:** 10.5812/atr.18950

**Published:** 2014-06-01

**Authors:** Mohammad Moazeni, Faramarz Mohammad Alibeigi, Masoud Sayadi, Ebrahim Poorya Mofrad, Soleiman Kheiri, Malihe Darvishi

**Affiliations:** 1 Department of Surgery, Faculty of Medicine, Shahrekord University of Medical Sciences, Shahrekord, IR Iran; 2 Kashani Hospital, Faculty of Medicine, Shahrekord University of Medical Sciences, Shahrekord, IR Iran; 3 Department of Anesthesiology, Faculty of Medicine, Shahrekord University of Medical Sciences, Shahrekord, IR Iran; 4 Social Health Determinants Research Center, Shahrekord University of Medical Sciences, Shahrekord, IR Iran

**Keywords:** Serum, Toxicity, Shotguns

## Abstract

**Background:**

Patients, who survived from shotgun injuries, often have some retained lead pellets in their bodies. Several cases of lead toxicity have been reported regarding these patients.

**Objectives:**

This study seeks to compare the serum lead level in patients who have retained lead pellets in their bodies with the control group.

**Patients and Methods:**

In this case-control study, we gathered the serum lead levels of 25 patients with some retained lead pellets in their bodies due to shotgun and 25 volunteers without similar lead exposure and compared them in view of the age, gender, and living place.

**Results:**

While the mean serum lead level in both groups was lower than the standard level (i.e. 40 µg/dL) , the mean ± SD of serum lead level were 29 ± 12.8 µg/dL and 25.3 ± 6.4 µg/dL in the case and control groups, respectively without any significant difference (P = 0. 30) . However, a positive relationship was seen between serum lead level, and the number of retained lead pellets (*r *= 0.447, P = 0. 025) .

**Conclusions:**

Although extensive surgery to remove the lead pellets is not recommended in patients injured with shotguns, those with many retained lead pellets in their bodies should be considered at risk for lead poisoning and monitored carefully.

## 1. Background

Lead is a heavy, soft and white to blue metal, which has been used in both households and industry, like lead paints. It can pollute air through industrial fumes and automobile exhausts and contaminate water, soil, and vegetables grown in lead-contaminated soil, and canned foods. Consequently, environmental lead exposure is widespread (1).

 Lead has many uses but is a potential toxin to humans too. Lead can enter into the body in different ways and creates harmful effects, especially in children (2). When the concentration of lead rises in the body, the initial symptoms of toxicity will appear as follows: anemia, lead paralysis and wrist drop, peripheral neuropathy often on the upper limbs, high uric acid level in the blood, anorexia, indigestion of food, diarrhea, and constipation (3, 4), skin and breast cancers (5), lymphoma (6), high serum cholesterol, LDL and HDL level, higher blood pressure and heart rate (7). Some significant symptoms of contact with lead are the blue lines on gums, loss of teeth at lower ages, and high blood pressure.

In developed countries, injuries resulted in firearms are considered as a common health problem which is not rare in Iran. One of the injuries is due to the shotgun that spread many lead pellets bearing target. The percentage of lead in lead pellets is more than 95%. When these lead pellets deliver into the body, lead releases gradually in the serum, depending on the location of the retained lead pellets in the body and the clinical condition of the patient (8).

There are only a few clear indications for bullet removal. These include bullets found in joints, CSF (cerebrospinal fluid), or the globe of the eye. Pellets leading to impingement on a nerve or a nerve root, and bullets lying in the lumen of a vessel, resulting in a risk of ischemia or remobilization, should be removed. Rare indications are lead poisoning caused by a fragment, and removal that is required for a medico-legal examination (9).

In these patients (in contrast to patients with clear indications for pellets removal) the retained lead pellets are not removed because of difficulty in operation and lack of study about pros and cons of extracting them. Therefore, most of the patients who survived from injuries of shotgun, have retained lead pellets in their bodies which may be absorbed by time and sometimes even after several decades of injuries, the serum lead level is high and may be the cause of toxicity and even patients' death. Most symptoms of toxicity are nonspecific, and the patients refer to physicians in a painful situation and even in this condition, diagnosis of lead toxicity delays or sometimes missed (8, 10, 11).

## 2. Objectives

On this subject, the studies are inadequate and the majority of studies are based on case studies (12-16) of toxicity with lead from retained lead pellets in the body that we cannot generalize it to all patients. Because of few studies about lead toxicity, injuries resulted in armies, especially shotguns, and rarity of these cases; the purpose of this study was to determine serum lead levels in patients with retained lead pellets in their bodies, compared to the control group, and if the serum lead level of the patients is higher than the standard level, the relationship between the number, location, and longevity of retained lead pellets with the serum lead level was determined.

## 3. Patients and Methods

This study was a case-control one that was approved by the Ethics Committee of the Shahrekord University of Medical Sciences. The participants included 25 patients enrolled as the case group and 25 volunteers enrolled as the matched control group based on convenience sampling. Cases were patients who complained of retained lead pellets in their bodies because they did not have indication for lead pellet removal, and had a file in Kashani hospital of Shahrekord from September 2004 to February 2011.

Retained lead pellets should be confirmed with plain film radiography. The control group was matched with the case group in view of the age, gender, and living place. Members of the control group were people who did not have lead pellets in their bodies, comorbid conditions, and other factors altering the blood lead level such as alcohol, hyperthyroid, acidosis, and the like, and interested in cooperating with the study.

Among patients who complained of retained lead pellets in their bodies over the last three months, 25 patients with these conditions were selected. In the next step, after calling on patients and providing explanations about our plan and the aim of this study, the questionnaire, including demographic information, effective factors on serum lead level and symptoms of toxicity with lead in their symptoms, signs and examinations, then blood sample was taken.

All subjects assigned written informed consent before they entered in the study. Venous blood (4 mL) was collected from the patients after cleaning the skin with cotton alcohol to dismiss any contamination with lead particles and also to prevent skin and soft tissue infections. From these 4 mL blood, 2 mL was dropped in a vessel of heparin sodium, and kept in the refrigerator freezer. Samples were sent to the Kimiapajooh Alborz laboratory to determine their serum lead level. In the laboratory, the lead level was determined by the atomic absorption spectrophotometer Zeeman correction graphite furnace (Perkin-Elmer 4100 PC) .

Two milliliter retained blood kept in a specified cell blood count (CBC) vessel, marked with the name of the patient and number, was sent to the laboratory to test. From control people, we collected 4 mL blood sample, of which 2 mL sample to measure serum lead levels and 2 mL to measure CBC were sent to the laboratory.

All patients were administrated a questionnaire to collect the following data: demographic information; history of comorbid conditions; and symptoms of lead toxicity. Also examiner evaluated and recorded the blood pressure, and signs and symptoms of lead toxicity. Our patients did not have any history of obvious lead exposure individuals who work in dyeing factories, except retained bullet lead pellets. Patients were also questioned on whether they experienced any of the following metabolic stresses in the past 30 days: surgery, alcohol abuse, illicit drug abuse, diabetic ketoacidosis, hyperthyroidism, infection, fracture, pregnancy, or lactation. Patients with retained lead pellets were also recorded other information regarding the date (s) of their shotgun injury and location (s) of the retained lead pellets.

 The mean ± standard deviation was used for descriptive statistics. The Kolmogorov-Smirnov test was used to evaluate continuous variables. Since the data did not have a normal distribution, the nonparametric test of Mann- Whitney and Spearman correlation coefficient were used for data analysis. Statistical significance was defined as P < 0.05 and analysis was performed using SPSS.

## 4. Results

Twenty-five subjects were studied in each group, 22 subjects (88%) were male in each group. The results of age, weight, systolic and diastolic blood pressure and hemoglobin and hematocrit levels in each group are presented in Table 1.

**Table 1.** The Results of Age, Weight, Systolic and Diastolic Blood Pressure, Hemoglobin and Hematocrit in the Case and Control Groups ^a^

Variable	Case	Control	P Value
**Age, y**	35.6 ± 13.3	35.4 ± 13.5	0.95
**Weight, kg**	71.3 ± 12.7	74.1 ± 17.3	0.524
**Systolic blood pressure, mmHg**	121.8 ± 11.3	120 ± 14.1	0.623
**Diastolic blood pressure, mmHg**	70.2 ± 19.4	76.4 ± 10.5	0.185
**Hemoglobin, g/dL**	15.17 ± 1.3	15.29 ± 1.4	0.763
**Hematocrit, %**	45.6 ± 3	45.7 ± 3	0.949

^a^ Data are presented as Mean ± SD.

Serum lead level in the case group was 14.5 to 71.5 µg/dL (mean: 29 ± 12.8) and in the control group was 14.5 to 39.7 (mean: 25.3 ± 6.4 µg/dL). The Mann-Whitney test did not demonstrate a significant difference in the serum lead level between the two groups (P = 0.30). Table 2 shows the hemoglobin and hematocrit levels, Lead pellets retention time in the body, and the numbers of lead pellets in the body in the case group.

**Table 2.** The Results of Hemoglobin, Hematocrit, Lead Pellets Retention Time in Month, and the Number Lead pellets in the Case Group ^a^

Variable	Min	Max	Results
**Hemoglobin, g/dL**	12.1	17.2	15.2 ± 1.3
**Hematocrit, %**	36.8	50.3	45.6 ± 3.1
**Lead pellet retention time, mo**	3	70	38.6 ± 16.8
**Number of lead pellets**	1	30	10.5 ± 9.7

^a^ Data are presented as Mean ± SD.

Results showed a positive significant relation between serum lead level and numbers of retained lead pellets in the body (*r* = 0. 447, P = 0. 025) . This test did not demonstrate any relation between hemoglobin, hematocrit and retention time of lead pellets in the body of the patients with their serum lead level. We did not find any significant relation between patients’ age or weight with their blood lead level. In the present study, symptoms of acute and chronic lead toxicity were checked in both groups, and we did not observe a significant difference between the case and control groups, and both groups did not show any symptoms of toxicity with lead.

## 5. Discussion

In the present study, the serum lead levels of the case group were 14.5 to 71.5 µg/dL with a mean and standard deviation of 29 ± 12.8. The serum lead levels of the control group were 14.5 to 39.7 with a mean and standard deviation of 25.3 ± 6.4. These static analytical results with Mann-Whitney tests did not show any differences in the serum lead level between the two groups, although the serum lead level in the case group is a little higher than that of the control group (P = 0.30) .

Farrell et al.in their study assessed serum lead levels of 15 patients with retained lead pellets in the body and 15 patients without lead pellets, which were matched in age, gender, race, and place of living. Mean serum lead level ± standard deviation for the case group was 17 ± 9.78 µg/dL and for the control group was 7 ± 3. 77µg/dL. They compared serum lead levels in two groups and found this difference statistically significant (12).

Nguyen et al. also investigated the serum lead levels of the patients with retained missiles in their bodies; in total, there were 120 participants in the case group and 120 in the control group. The serum lead level of five patients was higher than 20 µg/dL; the same finding was not observed in the control group. However, the mean serum lead level for the case and control groups were 6.71 and 3.16, respectively; this difference was statistically significant (10).

Lack of significant differences in serum lead level between the two groups of our study may be due to insolubility of lead pellets, which makes no threat of their systemic absorption for most patients. The exact procedure in the mobilization of the lead from pellets is still unclear. The location of the lead pellets in the body may also be important for redistribution of the lead; all the lead pellets in our patients were in the soft tissue and the extra-articular areas.

Although in our study, Spearman coefficient test showed no relation between retention time of lead pellets in body and the serum lead level of the patients, in another study conducted by McQuirter et al. (who assessed changes of serum lead level in an average of 0.3, 3.1, 18.7, 94.5, 188.3 and 394 days after entering of lead pellets), results showed that the lead level has been increased steadily after entering of lead pellets to the body up to 3 months and then stabilized (11). It might be due to granulation tissue formation around lead pellets that limit lead absorption. It is because of this minimum 3 months of retention time in our observed patients (between 3 to 70 months with the mean of 38.6 ± 16.8 months) that the serum lead level had reached to a stable level. Therefore, if there were any changes in patients in their first 3 months, we would not observe it in our patients.

In studies of Nguyen et al. long time retention of missiles in the body was not associated with a rising lead level (10). In our study, Spearman coefficient test showed a significant relation between the serum lead levels in the case group and the number of the retained lead pellets in the body (P = 0. 025, *r* = 0.447) . In other words, people who had more lead pellets in their bodies had higher serum lead levels. These results were similar to McQuirter et al. They also found that there was a significant relation between the number of remaining parts of the bullet, and the serum lead level (10).

In the present study, symptoms of lead toxicity were checked in both groups, and we did not observe any symptoms of toxicity with lead and significant difference between the case and control groups. In the study of Nguyen et al. no significant difference was also observed, which represented any symptoms of lead toxicity (10).

### 5.1. Lack of Symptoms of Lead Toxicity in Our Study

Symptoms of lead toxicity are nonspecific and sometimes hard to identify (13). A few lead fragments can remain in the body without any harm, and patients with retained lead pellets in their bodies may show their symptoms after 2 to 52 days (12). Harmful effects of lead in the body have not associated with a standard lead serum level so far. However, the serum lead level was considered to be higher than 40 µg/dL for toxicity.

 Elevated serum lead level, even if the measurement is accurate, does not prove lead intoxication but shows, instead, recent exposure or absorption of lead. Most experts believe that toxicity with lead is confirmed only by clinical symptoms; not specific serum lead level. In adults, some of the evidence shows subclinical effects with a low serum lead level, which its toxic harms present at the levels higher than 25 µg/dL (2). Intermittent nature of the symptoms in some cases makes them sometimes hard to identify.

 Scattered reports, summarized by Cagin et al. (14) and Dillman et al. (15) indicate that individuals with chronic lead poisoning develop clinical lead toxicity during periods of metabolic changes affecting bone (e.g. acidosis, hyperparathyroidism) or the nervous system (viral meningitis), or both (hyperthyroidism, acute infection). Although still controversial, alcohol has also been claimed to precipitate the symptoms of lead poisoning in patients with chronic exposure to lead (16, 17). With mobilization of the stored lead in bone, the soft tissue and serum levels increase, and clinical symptoms occur.

 In our study, the patients with these factors excluded from the study. Our study carried out in the highlands where people’s hemoglobin level is high. Despite the high serum lead level in some members of the case group, hemoglobin level in those patients is not low, that may be due to high altitude Patients with shotgun wounds are not recommended to be the subject of extensive surgery removing the lead pellets. However, patients with many retained lead pellets should be considered at risk for lead poisoning and monitored carefully.

### 5.2. Ethical Considerations

Ethical issues (Including plagiarism, Informed consent, misconduct, data fabrication and/or falsification, double publication and/or submission, redundancy, etc.) have been completely observed by the authors.
